# Supplementary material for: Midgut development in rat embryos using microcomputed tomography
Source: Commun Biol. 2021 Feb 12;4:190. doi: 10.1038/s42003-021-01702-4 (PMC7881192; doi:10.1038/s42003-021-01702-4)
Supplement: Supplementary file 2 — Supplementary Information. [file 42003_2021_1702_MOESM2_ESM.pdf]

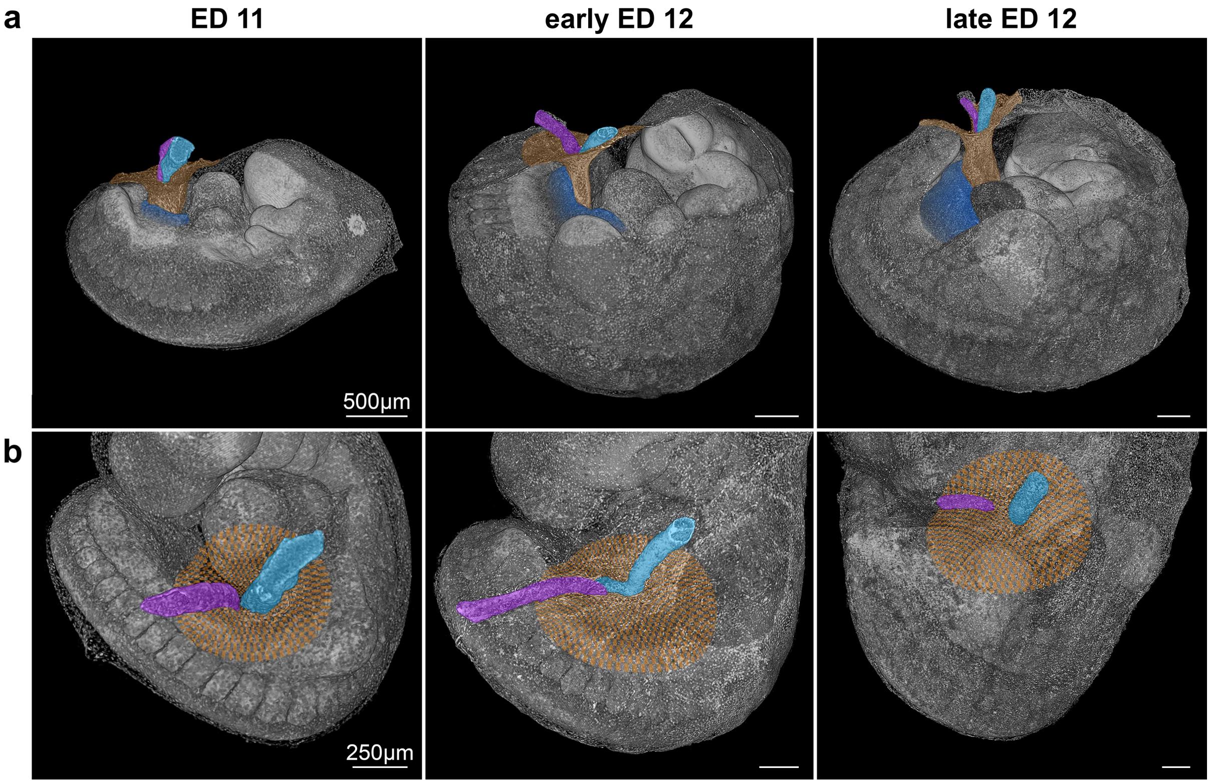


**Supplementary Fig. 1: Vitelline funnel development over time.** Representative reconstruction of embryos covered by the amniotic membrane with vitelline funnels from ED 11 to late ED 12. Reconstructions are shown from a skipped left view. Vessels and the vitelline funnel are colorized (umbilical vein: blue; vitelline artery: purple; vitelline vein: turquoise; vitelline funnel: orange). **a,** Partial removal of the amniotic membrane to allow the view to the funnel from inside. **b,** Top view of the funnel with an intact amniotic membrane.

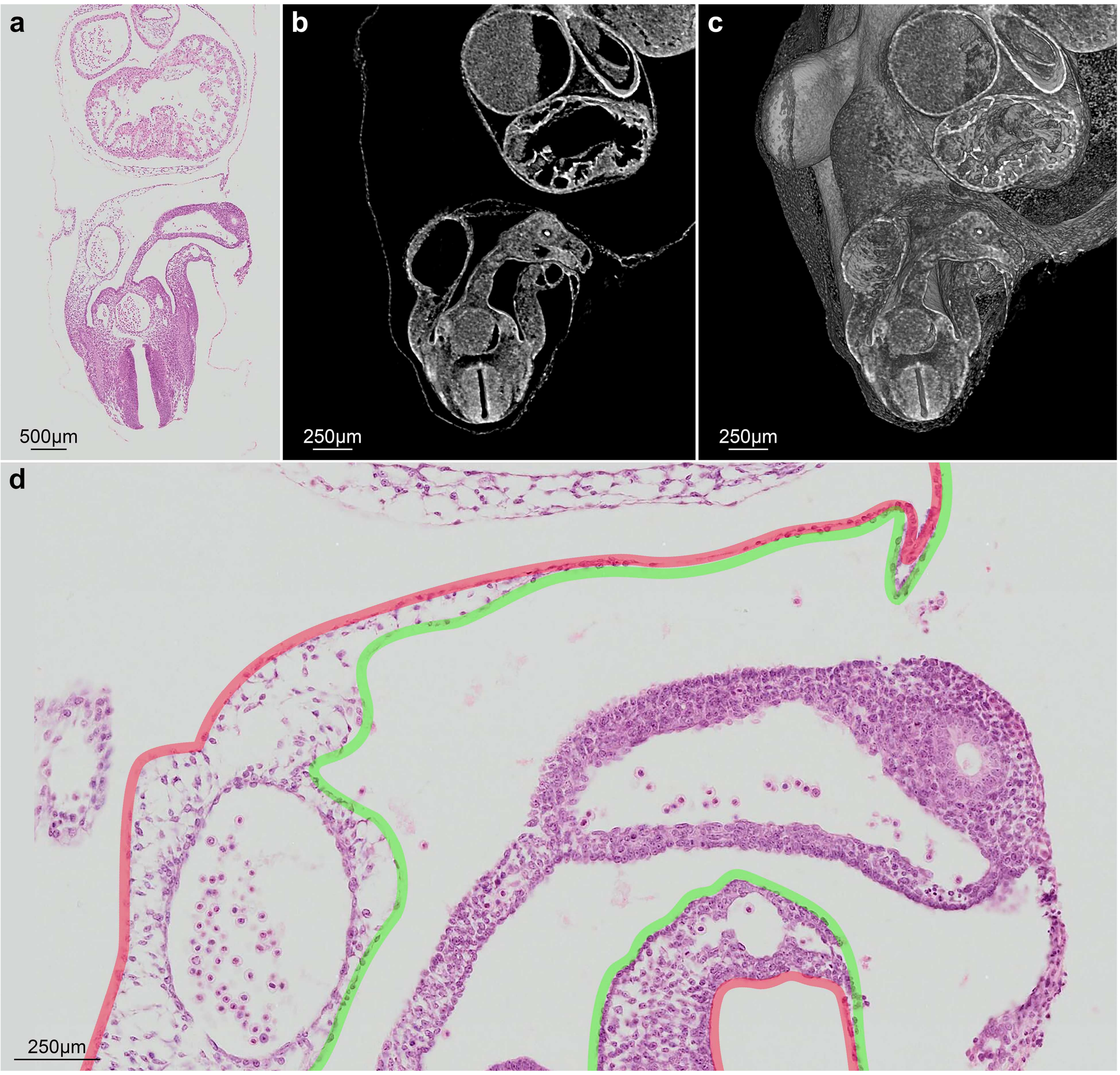


**Supplementary Fig. 2: Histological image of the vitelline funnel/compartment and its membranes. a,** Histological section of the vitelline compartment taken from: eHistology Atlas (http://www.emouseatlas.org/emap/eHistology/) (Plate 24c, Image f), http://www.emouseatlas.org/eAtlasViewer_ema/application/ema/kaufman/plate_24c.php?image=f (01.04.2020)[35]. **b,** Comparable section of an early ED 12 embryo of our collection. **c,** Same section as **b** including the reconstructed 3D embryo in the back. **d,** Magnification of **a,** with pseudocolored ectoderm (red) and mesoderm (green) layers of the amnion that separated close to the lateral body walls of the embryo. Adaptation of the picture according to the Creative Commons Attribution License.

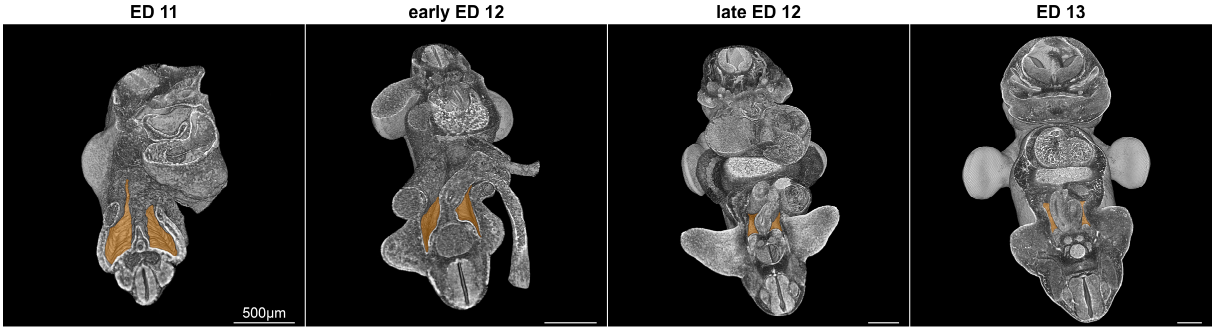


**Supplementary Fig. 3: Intraabdominal space.** Transverse sections of embryos from ED 11 to ED 13 showed a decrease in intraabdominal space over time. While at ED 11 and early ED 12, the abdominal space is theoretically capable of accommodating growing midgut parts, the first loop grows extraabdominally.

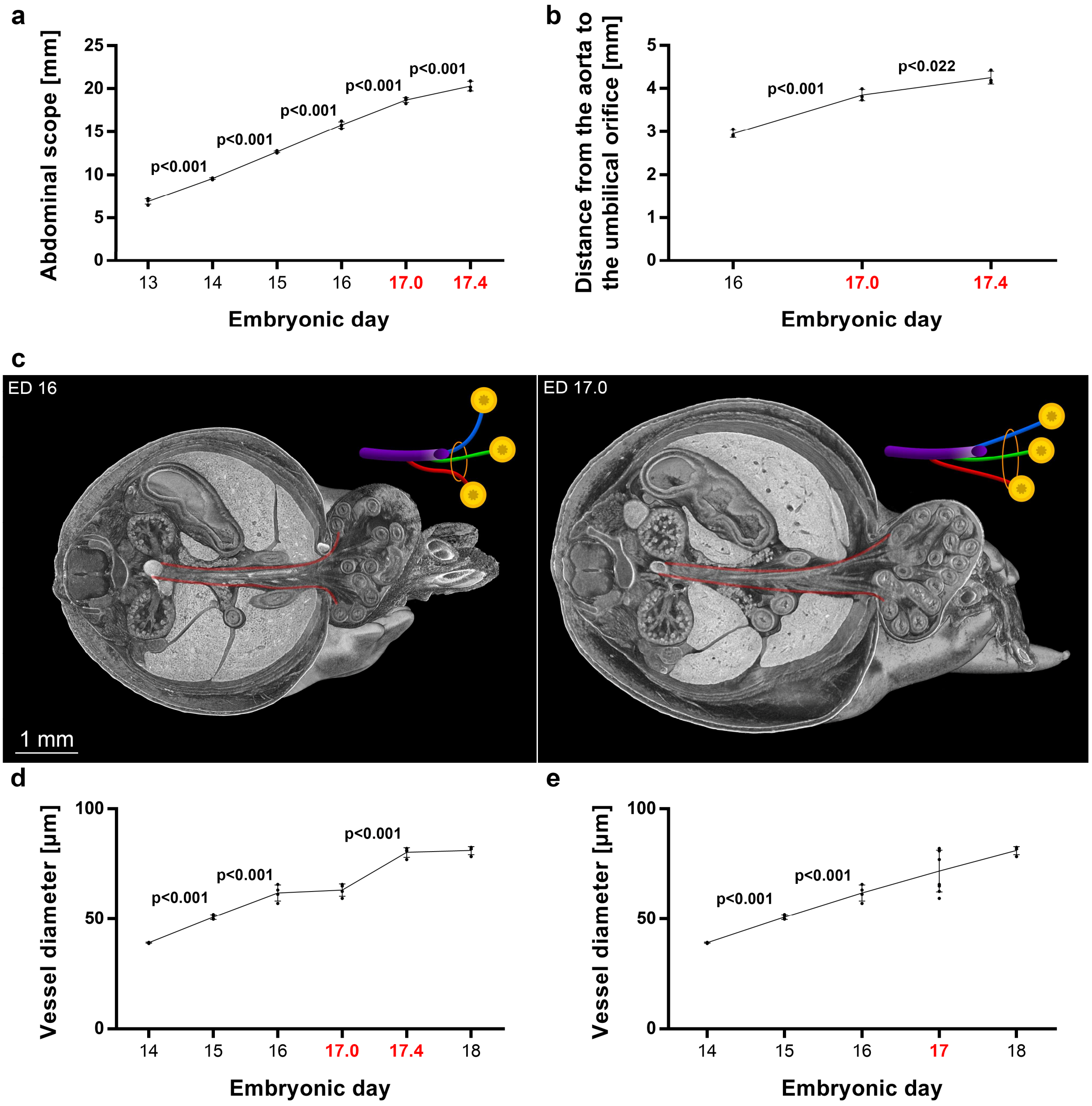


**Supplementary Fig. 4: Analysis of involved structures during the midgut shift. a,** Abdominal scopes from ED 13 to ED 17.4 over time are shown as single data points with SD (n = 3 – 4 animals). Effect sizes in Cohen´s f: ED 13 – ED 14: 1.47, ED 14 – ED 15: 1.69, ED 15 – ED 16: 1.71, ED 16 – ED 17.0: 1.59, ED 17.0- ED 17.4: 0.81. **b,** Distances between the aorta and the umbilical ring from ED 16 to ED 17.4 over time are shown as single data points with SD (n = 4 animals). Effect sizes in Cohen´s f: ED 16 – ED 17.0: 0.97, ED 17.0- ED 17.4: 1.25. **c,** Scaled transversal sections of embryos at ED 16 and ED 17.0 (before the midgut shift) showing the widening of the umbilical orifice caused by stretching of the SMA branches, which align to a cone-like structure

(indicated as red lines). The simplified drawings illustrate the behavior of the vessel bundles which supply three clusters of midgut loops. The assumed similar length of the three bundles and the positioning of their outlets on the SMA define the sequence of the shift (SMA: Purple, 1$^{st}$ cluster vessel: Red, 2$^{nd}$ cluster vessel: Green, 3$^{rd}$ cluster vessel: Blue, midgut loop cluster: yellow). **d,** Vessel diameters from ED 14 to ED 18 over time are shown as single data points with SD. We analyzed the situation before (ED 17.0) and after (ED 17.4) the midgut shift. Here, the shift itself resulted in a significant increase of vessel diameters (n = 4 animals). Effect sizes in Cohen´s f: ED 14 – ED 15: 2.39, ED 15 – ED 16: 2.26, ED 16 – ED 17.0: 0.28, ED 17.0- ED 17.4: 3.52, ED 17.4- ED 18: 0.17. **e,** Vessel diameters from ED 14 to ED 18 over time are shown as single data points with SD with no subdivision for ED 17, showing no significant increase from ED 16 to ED 18 due to a large standard deviation (n = 4 - 8 animals). Effect sizes in Cohen´s f: ED 14 – ED 15: 1.87, ED 15 – ED 16: 1.77, ED 16 – ED 17: 1.85, ED 17 – ED 18: 1.75. To0 highlight the subdivision of ED 17 it was marked in red.

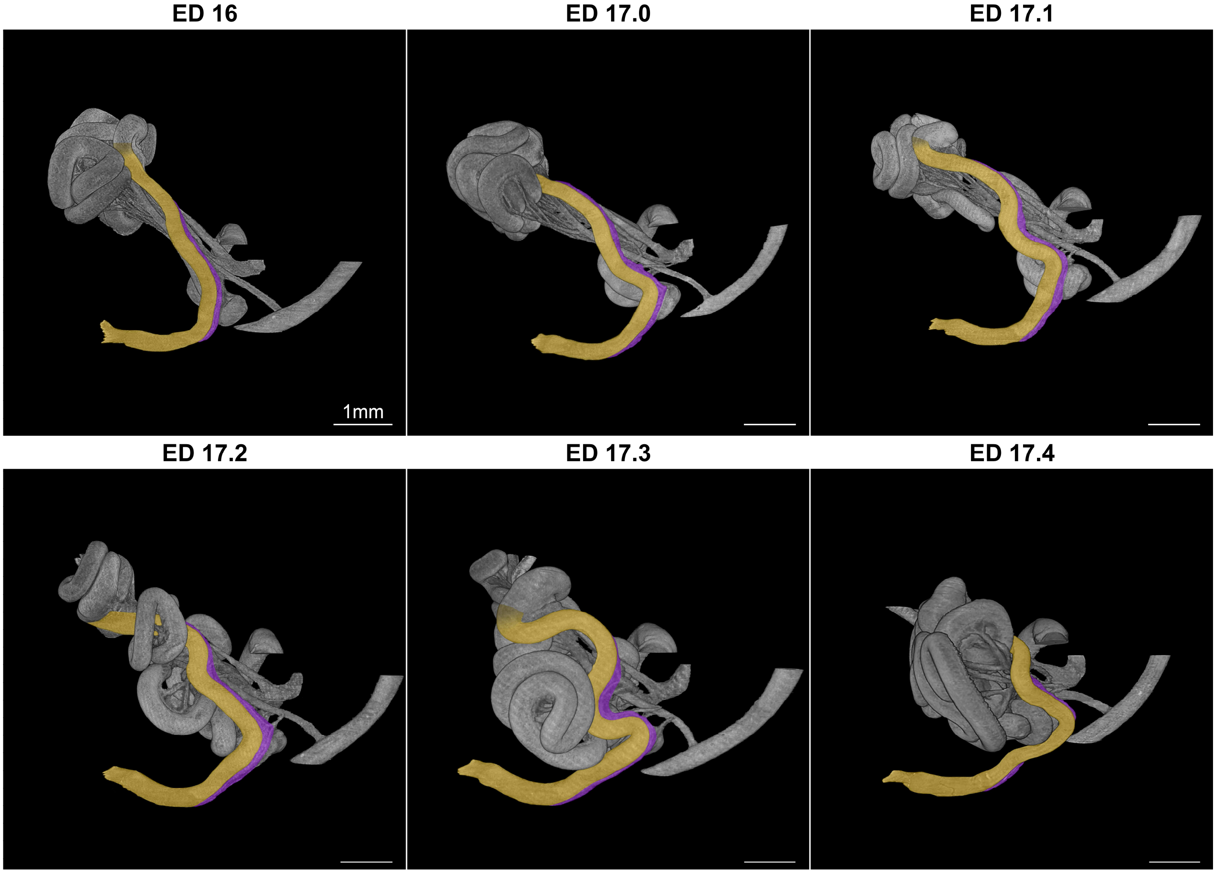


**Supplementary Fig. 5: Behavior of the colon during the intestinal shift.** Representative reconstructions of the intestines with the supplying blood vessels from ED 16 to ED 17.4 from the left side (Colon: yellow; colonic vessel: purple).

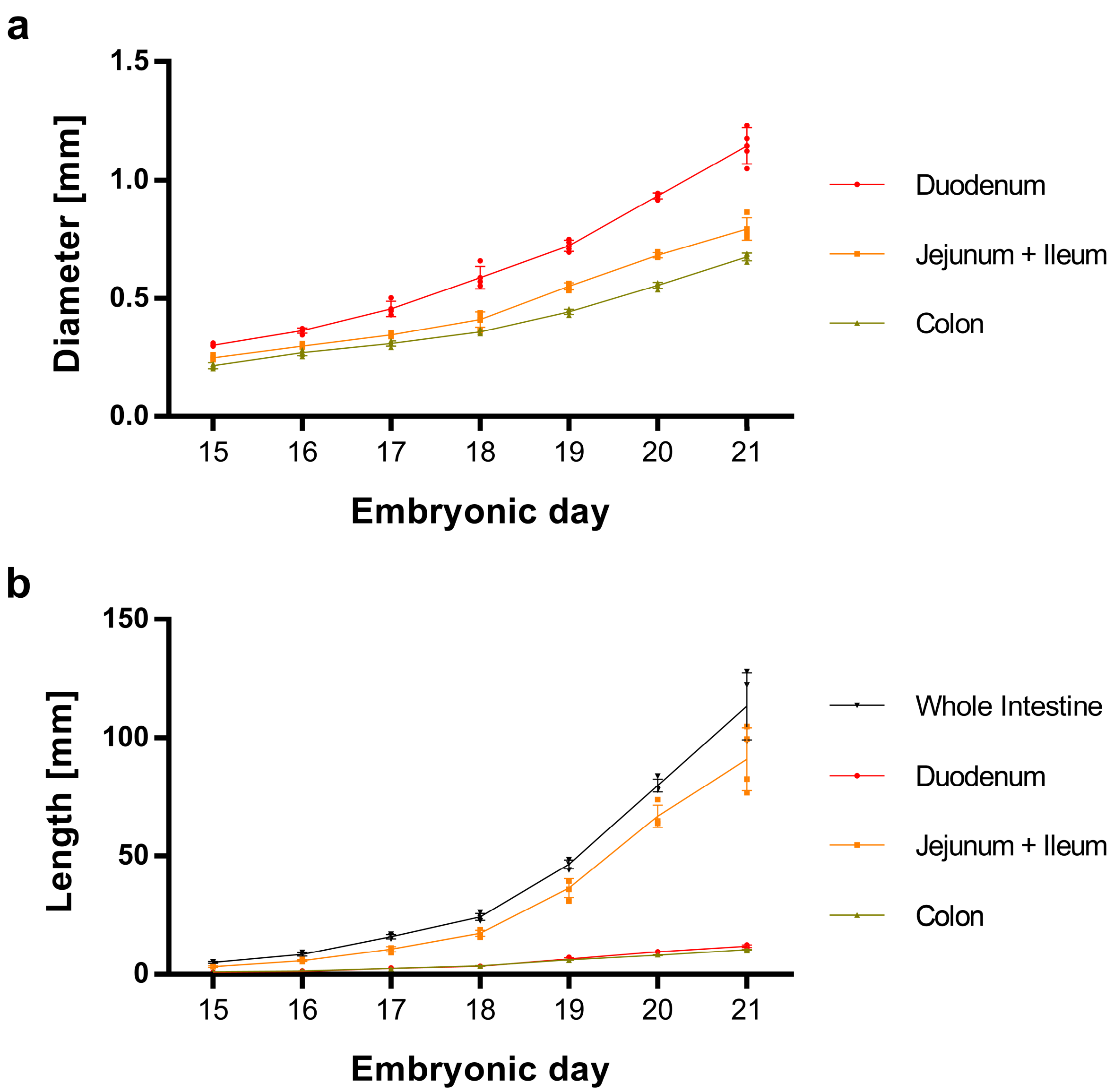


**Supplementary Fig. 6: Diameter and calculated length of the intestinal segments.**

**a,** Direct measurement of the diameters of each intestinal segment from ED 14 to ED 21. **b,** Calculated length of the intestinal segments by volume (V) and diameter (D). (V / ((D / 2)$^2$ π)).

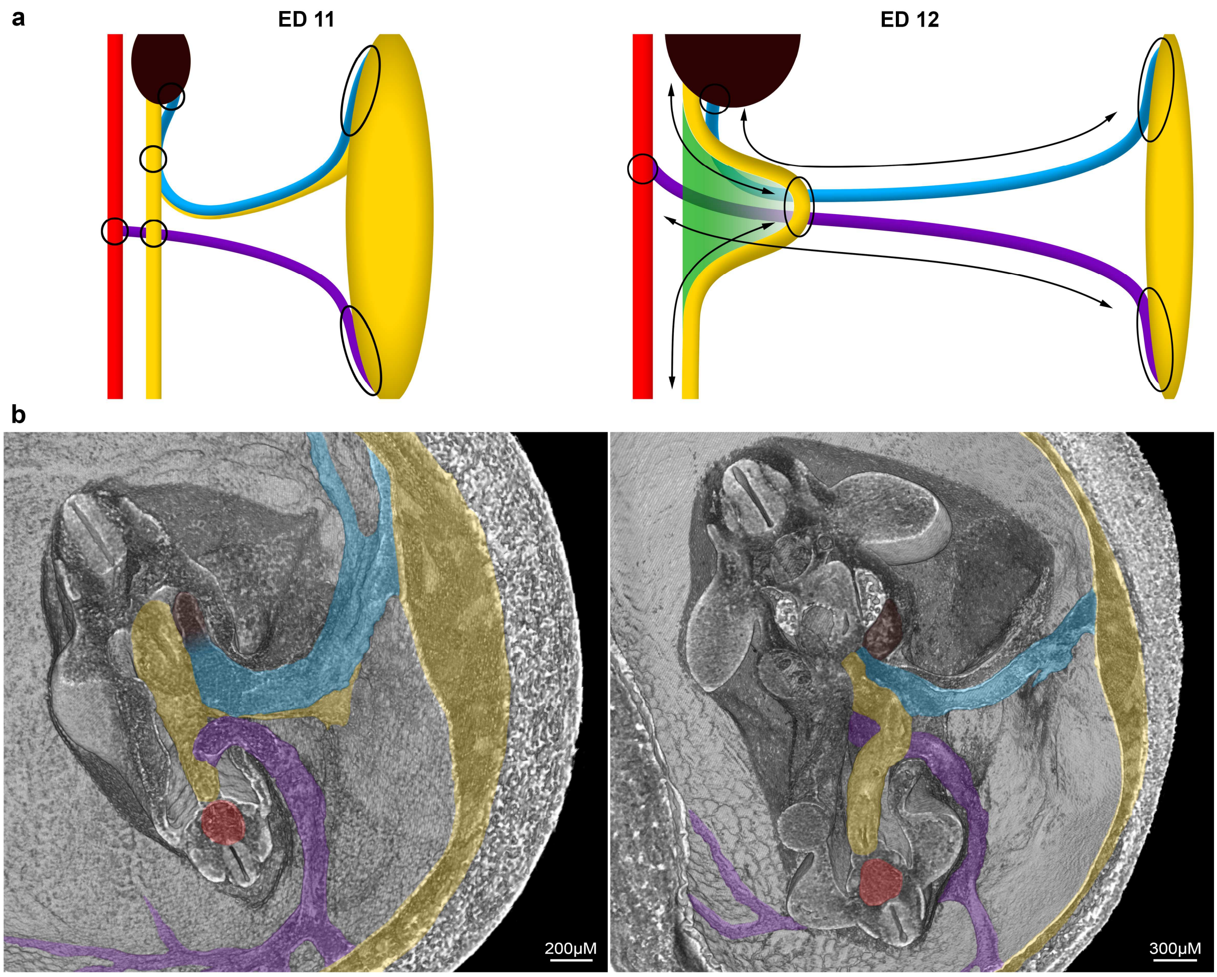


**Supplementary Fig. 7: Implications of vitelline structures on the formation and direction of the first midgut loop. a,** Schematic drawing of the vitelline structures, the developing midgut, and the yolk sac (vitelline artery: purple, vitelline vein: turquois, midgut, ductus omphaloentericus, yolk sac: yellow, and emerging mesentery: green). The vitelline vessels connect the yolk sac to the aorta and the liver respectively, and are also attached to the midgut. The connection to the yolk sac serves as an anchoring point. A growth of the vitelline vessels together with the midgut leads to the formation of the first loop, by directing the tip of the midgut loop outside and to the left of the embryo. The attachment points are marked with black circles. The black arrows indicate growth. **b,** Corresponding scaled reconstructions of embryos at ED 11 and ED 12.

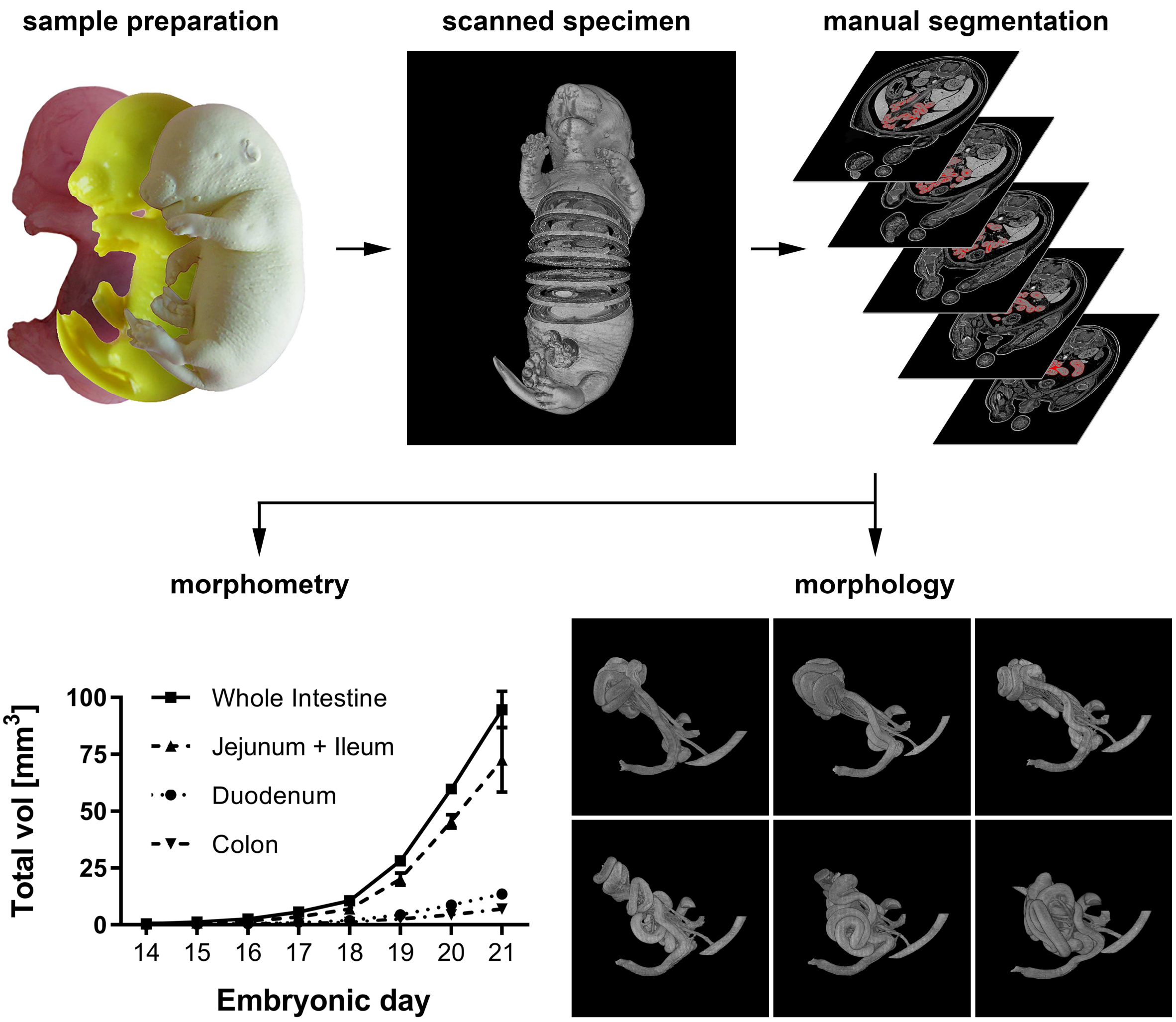


**Supplementary Fig. 8: Schematic workflow.** Rat embryos of gestational ages from ED 10 to ED 21 were harvested, fixed in Bouin's solution and subsequently dried according to the critical point drying method. After µCT scanning, regions of interest were marked manually. The reconstructed tissues were used for morphometric and morphologic evaluation.
