# Supplementary material for: Midgut development in rat embryos using microcomputed tomography
Source: Commun Biol. 2021 Feb 12;4:190. doi: 10.1038/s42003-021-01702-4 (PMC7881192; doi:10.1038/s42003-021-01702-4)
Supplement: Supplementary file 3 — Description of Additional Supplementary Files [file 42003_2021_1702_MOESM3_ESM.pdf]

Description of Additional Supplementary Files

Supplementary movie 1: Rat embryo in uterus at ED 11

Labeled 3D µCT video of a rat embryo at ED 11 using CTvox® (Bruker microCT, Kontich, Belgium) software. The video gives an overview of this developmental stage.

Supplementary movie 2: Rat embryo in uterus at early ED 12

Labeled 3D µCT video of a rat embryo at early ED 12 using CTvox® (Bruker microCT, Kontich, Belgium) software. The video gives an overview of this developmental stage.

Supplementary movie 3: Rat embryo in uterus at late ED 12

Labeled 3D µCT video of a rat embryo at late ED 12 using CTvox® (Bruker microCT, Kontich, Belgium) software. The video gives an overview of this developmental stage.

Supplementary Video 4: Ventral View Midgut Development ED12-ED13

Morphing video of the midgut from early ED12 to ED 13 generated by interpolating a sequence of images obtained by µCT using FotoMorph software (Version 13.9.1). Ventral view.

Supplementary Video 5: Lateral View Midgut Development ED 12-ED13

Morphing video of the midgut from early ED12 to ED 13 generated by interpolating a sequence of images obtained by µCT using FotoMorph software (Version 13.9.1). Lateral view.

Supplementary Data 1: Raw data of measurements
