# Supplementary material for: Midgut development in rat embryos using microcomputed tomography
Source: Commun Biol. 2021 Feb 12;4:190. doi: 10.1038/s42003-021-01702-4 (PMC7881192; doi:10.1038/s42003-021-01702-4)
Supplement: Supplementary file 10 — Reporting Summary [file 42003_2021_1702_MOESM10_ESM.pdf]

nature research

Corresponding author(s): Marco Ginzel

Last updated by author(s): Nov 16, 2020

Reporting Summary

Nature Research wishes to improve the reproducibility of the work that we publish. This form provides structure for consistency and transparency in reporting. For further information on Nature Research policies, see our Editorial Policies and the Editorial Policy Checklist.

Statistics

For all statistical analyses, confirm that the following items are present in the figure legend, table legend, main text, or Methods section.

n/a	Confirmed	
☐	☒	The exact sample size (n) for each experimental group/condition, given as a discrete number and unit of measurement
☐	☒	A statement on whether measurements were taken from distinct samples or whether the same sample was measured repeatedly
☐	☒	The statistical test(s) used AND whether they are one- or two-sided<br>*Only common tests should be described solely by name; describe more complex techniques in the Methods section.*
☒	☐	A description of all covariates tested
☐	☒	A description of any assumptions or corrections, such as tests of normality and adjustment for multiple comparisons
☐	☒	A full description of the statistical parameters including central tendency (e.g. means) or other basic estimates (e.g. regression coefficient) AND variation (e.g. standard deviation) or associated estimates of uncertainty (e.g. confidence intervals)
☐	☒	For null hypothesis testing, the test statistic (e.g. F, t, r) with confidence intervals, effect sizes, degrees of freedom and P value noted<br>*Give P values as exact values whenever suitable.*
☒	☐	For Bayesian analysis, information on the choice of priors and Markov chain Monte Carlo settings
☒	☐	For hierarchical and complex designs, identification of the appropriate level for tests and full reporting of outcomes
☐	☒	Estimates of effect sizes (e.g. Cohen's d, Pearson's r), indicating how they were calculated

Our web collection on statistics for biologists contains articles on many of the points above.

Software and code

Policy information about availability of computer code

Data collection	NRecon, version 1.7.0.4; Bruker, Kontich, Belgium/ CTAn®, version 1.16.1.0; Bruker
Data analysis	SPSS, version 26; IBM®, Armonk, NY, USA/ graphical data analysis: CTvox, version 3.0; Bruker, Kontich, Belgium

For manuscripts utilizing custom algorithms or software that are central to the research but not yet described in published literature, software must be made available to editors and reviewers. We strongly encourage code deposition in a community repository (e.g. GitHub). See the Nature Research guidelines for submitting code & software for further information.

Data

Policy information about availability of data

All manuscripts must include a data availability statement. This statement should provide the following information, where applicable:

- Accession codes, unique identifiers, or web links for publicly available datasets
- A list of figures that have associated raw data
- A description of any restrictions on data availability

The micro-CT datasets consist of single images in .png format. The images in each set can be combined into three-dimensional structures by using appropriate software such as CTvox, CTAn (both software from Bruker, Kontich, belgium) or Amira (software from Thermo Fisher Scientific, Waltham, MA, USA). The individual images can also be viewed with any graphic program. Our datasets are openly available in Publissio ZB MED Information Centre of Life Science. All measured raw data is present in the Supplementary Data.

Field-specific reporting

Please select the one below that is the best fit for your research. If you are not sure, read the appropriate sections before making your selection.

☒ Life sciences ☐ Behavioural & social sciences ☐ Ecological, evolutionary & environmental sciences

For a reference copy of the document with all sections, see nature.com/documents/nr-reporting-summary-flat.pdf

Life sciences study design

All studies must disclose on these points even when the disclosure is negative.

Sample size	This study is primarily based on morphological changes during embryogenesis and manual segmentation of embryonic structures is highly time consuming. To provide a minimal statistic for measured values, a sample size of 4 animals per age group from ED12 onwards was considered sufficient.
Data exclusions	No data was excluded.
Replication	The reproducibility is given, as two embryos per litter and two litters per age group were processed separately and investigated, if applicable (One exception is the shown return of the midgut loops from the umbilicus to the abdominal cavity). Additionally, age matched embryos showed similar patterns and measured values.
Randomization	Randomization was not relevant to our study as only one group was investigated
Blinding	Blinding was not relevant to our study as only one group was investigated

Reporting for specific materials, systems and methods

We require information from authors about some types of materials, experimental systems and methods used in many studies. Here, indicate whether each material, system or method listed is relevant to your study. If you are not sure if a list item applies to your research, read the appropriate section before selecting a response.

Materials & experimental systems

n/a	Involved in the study	
☒	☐	Antibodies
☒	☐	Eukaryotic cell lines
☒	☐	Palaeontology and archaeology
☐	☒	Animals and other organisms
☒	☐	Human research participants
☒	☐	Clinical data
☒	☐	Dual use research of concern

Methods

n/a	Involved in the study	
☒	☐	ChIP-seq
☒	☐	Flow cytometry
☒	☐	MRI-based neuroimaging

Animals and other organisms

Policy information about studies involving animals; ARRIVE guidelines recommended for reporting animal research

Laboratory animals	Spraque Dawlay rat embryos regardless of their sex were used.
Wild animals	This study did not involve wild animals.
Field-collected samples	This study did not involve samples collected from the field.
Ethics oversight	Animal care and experimental procedures were approved by the institutional review board in Saxony, Germany (state directorate Saxony, Referat 25, veterinary and food monitoring, Braustraße 2, 04107 Leipzig. Proposals: T14/15, T44/16, T13/18)

Note that full information on the approval of the study protocol must also be provided in the manuscript.
